# Dengue 3 Epidemic, Havana, 2001

**DOI:** 10.3201/eid1004.030271

**Published:** 2004-04

**Authors:** Otto Peláez, María G. Guzmán, Gustavo Kourí, Raúl Pérez, José L. San Martín, Susana Vázquez, Delfina Rosario, Regla Mora, Ibrahim Quintana, Juan Bisset, Reynel Cancio, Ana M Masa, Osvaldo Castro, Daniel González, Luis C. Avila, Rosmari Rodríguez, Mayling Alvarez, Jose L. Pelegrino, Lídice Bernardo, Irina Prado

**Affiliations:** * Centro Provincial de Higiene y Epidemiología de Ciudad Habana, Habana, Cuba; † Instituto Medicina Tropical “Pedro Kourí”; ‡ Viceministerio para la Higiene y la Epidemiología, Habana, Cuba

**Keywords:** Dengue, Dengue Hemorrhagic Fever, Cuba, Dengue 3, Havana

## Abstract

In June 2001, dengue transmission was detected in Havana, Cuba; 12,889 cases were reported. Dengue 3, the etiologic agent of the epidemic, caused the dengue hemorrhagic fever only in adults, with 78 cases and 3 deaths. After intensive vector control efforts, no new cases have been detected.

In 1994, after 17 years of absence, Nicaragua, Panama, and Costa Rica reported the reintroduction of dengue 3 virus in the region (*1*,*2*). The last isolation of this serotype occurred in 1977–1978 in Puerto Rico and Colombia (*1*,*2*). Dengue 3 has been related to dengue fever (DF) and dengue hemorrhagic fever (DHF) epidemics. In 7 years the virus has disseminated first to Central-American countries, and later to Mexico, Caribbean countries, and more recently, to South America (*3*). This virus is genetically different from the dengue 3 strain previously isolated in the Americas and belongs to the same genotype as the virus that caused DHF epidemics in Sri Lanka and India (subtype III) (*4*). Currently, it is believed that millions of persons in the American region are considered to be at risk of dengue infection.

Previously, larger epidemics in Cuba were associated with dengue 1 in 1977 and with dengue 2 in 1981. Both epidemics affected the entire country, producing more than 500,000 and 300,000 dengue cases, respectively. More than 10,000 cases of DHF causing 158 deaths were reported in 1981. From 1982 to 1996, no dengue transmission was reported. In 1997, a dengue 2 epidemic was reported in the municipality of Santiago de Cuba, located in the eastern part of the country (*5*). In September 2000, a small outbreak of dengue was detected in Havana City; 138 cases of dengue fever (DF) were confirmed at that time, and both dengue 4 and dengue 3 viruses were isolated; the outbreak ended by December (*6*,*7*).

## The Study

Havana is the capital city of the Republic of Cuba with 15 municipalities, 2,193,848 inhabitants, and a population density of 3,040/km^2^. Located in the north of the country, it covers an area of 720.84 km^2^ and has an annual average temperature of 25°C. La Habana province surrounds Havana City on the east, west, and south. House indexes (percentage of houses with at least one infested container) of 0.05 to 0.91 were reported from 1997 to 2001. In July 2001, house indexes at the municipalities of the city varied from 0.2 to 1.5; however, higher figures were observed at health areas and blocks. These data demonstrate that transmission risk must be assessed in more numerous, smaller geographic areas. The entomologic surveillance and vector control activities involved 4,796 workers; 3,278 family doctors’ offices (one family doctor per 120 families and 600 inhabitants) and 81 health areas constitute the primary health care system, and 23 hospitals comprise the second and third levels.

Once the Santiago de Cuba epidemic was detected in January 1997 (*5*,*8*,*9*), an active dengue surveillance system was established throughout the country. Specifically in Havana City, the surveillance was directed at detecting dengue transmission by studying patients with undifferentiated fever and patients with suspected dengue (patients with fever and two or more symptoms of DF such as myalgia, arthralgia, headache, and rash).

A serum sample for dengue immunoglobulin (Ig) M detection was collected 5 days after onset of fever. IgM studies were conducted first at the laboratory of the Centro Provincial de Higiene y Epidemiologia de Ciudad Habana (CPHE-CH) by using the ultramicro-enzyme-linked immunosorbent assay (ELISA) for dengue IgM detection (*10*). Positive samples were confirmed at the national reference center, the Tropical Medicine Institute (IPK) by an IgM capture ELISA (*11*). A comprehensive study from clinical, epidemiologic, and entomologic perspectives was conducted at those health areas where case-patients were found; a second serum sample was collected 2–3 weeks after illness onset to demonstrate the antibody seroconversion or a 4-fold increase in antibody titer (*12*). The Table shows the total number of serum samples studied from 1997 to 2002.

**Table:** Total serum samples from Havana City analyzed at both the provincial (CPHE-CH) and national level (IPK), 1999–2002^a^

Y	CPHE-CH	IPK
1997	807	9,538
1998	1,377	4,794
1999	4,166	10,012
2000	39,335	19,752
2001	65,770	38,513
2002^b^	11,302	13,304
Total	122,757	95,913

^a^ CPHE-CH,Centro Provincial de Higiene y Epidemiologia de Ciudad Habana; IPK, Tropical Medicine Institute. ^b^ Through March 2002.

In June 29, 2001, a confirmed dengue case was reported to the national health authorities. The index case had an onset date of June 16. The index case-patient was a 68-year-old white woman who lived in the “26 de Julio” health area of the Playa municipality; she had no history of travel outside the country. The “26 de Julio” health area was a residential location with a noncontinous water supply (it received water every 2 days). The house index was 2.1. Many persons from dengue-endemic countries lived in the area, and many boarding houses also characterized this area.

Within 2 weeks, 20 additional DF cases were serologically confirmed. A retrospective seroepidemiologic study was conducted in a radius of 1 km^2^ around the index patient to look for any patients with suspected dengue or undifferentiated fever; 312 febrile patients, and 14 suspected DF patients were found; however, DF was confirmed by serologic studies in 4 of them. All 4 case-patients had dengue IgM and high titers of IgG dengue antibodies. Epidemiologic studies of these patients showed that the first case occurred in late May or early June. The primary case-patient was a 53-year-old white man from the same health area as the index patient.

Once transmission was confirmed, a proactive dengue surveillance program was established, based on information from family doctors. Virologic and molecular surveillance demonstrated that dengue 3 was the etiologic agent of the epidemic. Ninety-one dengue 3 isolates were obtained from samples collected at various times during the epidemic.

**Figure 1:**
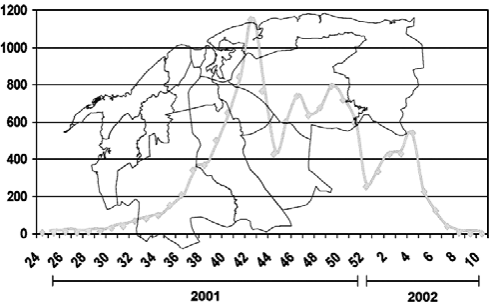
Dengue confirmed case notification according to onset of fever

**Figure 2:**
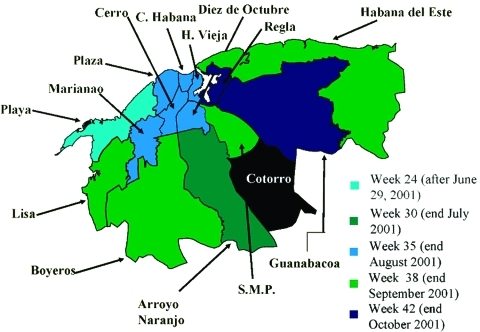
Extension of the epidemic in Havana City, 2001-2002

Considering the active surveillance and that specimens from all identified clinical case-patients were studied by serologic or virologic methods, the figure of confirmed cases is very close to the total number of dengue clinical cases of the epidemic. All confirmed case-patients were notified of their illness. Figure 1 shows the histogram of the epidemic, and Figure 2 shows the municipality distribution and the date of confirmed transmission in the city. By week 30 (July) new cases were detected in the Arroyo Naranjo Municipality, and by the end of October (week 42), almost all municipalities had reported dengue transmission.

The wide clinical spectrum of dengue was established in the Pan American Health Organization (PAHO)/World Health Organization (WHO) guidelines (*13*). Because of the detection of dengue transmission in the city, the existence of the primary health system, and the strong dengue surveillance system that included laboratories with appropriate technology for serologic diagnosis, we decided to extend the clinical, epidemiologic, and laboratory surveillance to the study of almost all undifferentiated fever cases and those patients with a compatible classic dengue picture. A house-by-house survey for febrile cases and dengue suspected cases was performed in Havana City by the family doctors. As a result, 72,162 cases (41,830 undifferentiated fever and 30,332 dengue suspected cases) were epidemiologically, clinically, and serologically studied. Dengue infection was confirmed in 12,889 (17.86%) of the total cases. Of patients with confirmed cases, 1,660 (12.9%) were children and 11,229 were adults (87.1%); 52.4% were female and 47.6% were male. DHF was diagnosed in 78 patients, all adults (16–64 years of age). The main signs and symptoms detected in patients with confirmed dengue cases at the time of hospital admission were fever, 100%; headache, 89%; retrorbital pain, 59.2%; arthralgia, 59.4%; myalgia, 35.2%; and rash, 28.1%. Other symptoms such as cough, diarrhea, nausea, and vomiting were observed in 21.2% of case-patients.

The peak of the epidemic occurred in October and the highest number of cases occurred on October 20 (241 confirmed cases); 1,150 cases were confirmed by week 42 (October 14–20).

The onset of symptoms of the last two case-patients occurred by February 22, 2002. The epidemic was considered controlled 36 days later with confirmation that no possibility of transmission existed. Case fatality rate was 3.8% among patients with DHF/dengue shock syndrome (DSS). Mortality rate was 0.13/100,000 inhabitants and morbidity rate was 59.2/ 10,000 inhabitants.

## Conclusions

After the first cases were detected, all patients with suspected dengue and those who were severely ill, or those classified as having DHF/DSS were hospitalized, all adults at the IPK hospital and all children at the Aballi and Cerro Pediatric Hospitals. In total, 4,184 patients were hospitalized, 3,197 adults and 987 children. By the end of the epidemic in January 2002, a broad hospitalization policy was established in areas free of vector, *Aedes aegpyti* mosquitoes, (all febrile and dengue suspected case-patients were hospitalized or treated at home with daily visits by the family doctor) (*13*). Anatomopathologic and histopathologic studies were conducted in all fatal cases.

The vector control strategy had two phases: the first started as soon as the transmission was detected and restricted the number of cases and geographic extension of the epidemic (the risk of expansion of the epidemic was high because of the vector indexes in Havana City and other provinces).The second phase, called the Intensive Campaign, started at the beginning of January 2002 and interrupted transmission and, consequently, lowered the risk of dengue endemicity in approximately 70 days. The Campaign was based on the principles of dengue control established by the PAHO Guidelines (*13*) with the involvement of the whole community (the head of state, governmental and political bodies at all levels, householders, community organizations, etc.) The objectives of the Intensive Campaign were to control the vector and interrupt dengue transmission. Massive environmental management and sanitation efforts, the elimination of breeding sites, and the elimination of adult mosquitoes were also carried out. These activities were accompanied by extensive efforts to mobilize the community, a strong program of quality control, the active media involvement, and the repositioning of tanks and different water containers. From a house index of 0.49 at the beginning of the Intensive Campaign, this figure diminished to 0.01 by March 4. The intensive active surveillance and the hospitalization of all febrile patients and all patients thought to be infected with dengue were crucial in order to reduce the dengue transmission.

At present, after 17 months since the last dengue case, strong surveillance is maintained by the six regional laboratories and the national reference center, and no additional cases have been reported. These data indicate that endemicity was avoided. Efforts are being made to eliminate *Ae. aegpyti* in a regional situation in which the disease has caused unprecedented numbers of cases of DF and DHF (1,015,420 dengue cases and 14374 dengue hemorrhagic fever, with 225 deaths have been reported to PAHO) (data provided by Jorge Arias, WHO American regional office). As has been stated in the PAHO resolution approved by the PanAmerican Health Assembly in September 2002 (*14*), a concerted action at a regional level is urgently needed.
